# A systematic literature review of data envelopment analysis implementation in agriculture under the prism of sustainability

**DOI:** 10.1007/s12351-023-00741-5

**Published:** 2023-02-15

**Authors:** Leonidas Sotirios Kyrgiakos, Georgios Kleftodimos, George Vlontzos, Panos M. Pardalos

**Affiliations:** 1 Department of Agriculture, Crop Production and Rural Environment, University of Thessaly, Fytoko, 38446 Volos, Greece; 2 Mediterranean Agronomic Institute of Montpellier (CIHEAM-IAMM), 34093 Montpellier, France; 3 Department of Industrial and Systems Engineering, University of Florida, 401 Weil Hall, Gainesville, FL 32611-6595 USA

**Keywords:** Data envelopment analysis, DEA, Agriculture, Efficiency, Sustainability, Review

## Abstract

Safeguarding natural resources and energy is essential to ensure food security for future generations. Given the increase of published papers in the agricultural field applying Data Envelopment Analysis (DEA), this review seeks to address the special requirements of this methodology when implemented in the agricultural sector as well as to classify papers under sustainability aspects (economic, environmental, social). More specifically, 120 papers from Scopus and Web of Science databases were included in this review by using PRISMA methodology, and they were tested in the following groups (i) General information, (ii) DEA implementation, (iii) DEA extensions, (iv) Data type, (v) Data collection and processing, and (vi) Sustainability dimensions. Results indicate that there is a great need for weights use when performing DEA in the agricultural sector, to acquire results with greater explanatory power. Moreover, systematic data collection of multiple factors could lead to the implementation of complex methodologies, providing feasible solutions to the involved stakeholders. Lastly, the social aspect is the least represented dimension out of the three aspects of sustainability, indicating the need for the integration of social factors in such analyses, especially when DEA is used to create a policy framework in a specific area.

## Introduction

Food security, overpopulation, and conservation of natural resources are the biggest challenges for today's agriculture (Calicioglu et al. 2019). In addition, the global trend towards adopting Sustainable Development Goals (SDGs) (United Nations 2015) has not left the agricultural sector unaffected, as the same principles will have to be integrated into this sector for (United Nations 2015) sustainable agriculture (European Commission 2015).

Although the search for «sustainability» term shows a slight increase from 2014 to 2021 for general Google users, there is a rapid increase in searching for the term in the academic community of 160% for the same period, verifying the effort of researchers to find solutions or methodologies to achieve the globally accepted sustainable development goals (Google Trends 2021). Taking into consideration the need to provide food for an ever-growing population with an inexhaustible number of available resources, leads humanity to the establishment of new systems or the invention of new technologies which can produce the same amount of output using the least possible energy and resources. In other words, for ensuring sustainability in agriculture, the efficiency of existing systems needs to be increased. On operational terms, this means that either production levels should remain at the same levels with the need for inputs to be decreased, or output should be increased, given the inputs used. With this goal reassurances can be provided that future generations will have equal opportunities to access energy and natural resources.

Following the above line of reasoning, efficiency analyses can contribute to quantifying losses and highlight weak points on production processes in the agricultural sector, to minimize the exploitation of natural resources while producing adequate amounts of feed and food. Efficiency measurement can be achieved by using either parametric (e.g. Stochastic Frontier Approach -SFA (Aigner et al. 1977)) or non-parametric approaches such as DEA (Charnes et al. 1978). SFA is capable of distinguishing noise from inefficiency, however, DEA includes noise in its final results (Lampe and Hilgers 2015). Moreover, SFA is not so sensitive to outliers as DEA, due to the fact that SFA is based on regression models, while DEA computations are based on linear programming principles. Removal of outliers is a crucial stage for data preparation when performing DEA, which may end up in a false interpretation of the results if neglected (Sarkis 2007). On the other hand, DEA is mostly used in the agricultural sector, due to the fact that it can handle multiple inputs and outputs, in contrast with conventional SFA models, which can handle single input or output and multiple inputs or outputs. DEA also does not need any prior assumption about inputs and outputs relationship, compared with SFA, a decision that may lead to uncertain results (Watto and Mugera 2019).

**Fig. 1:**
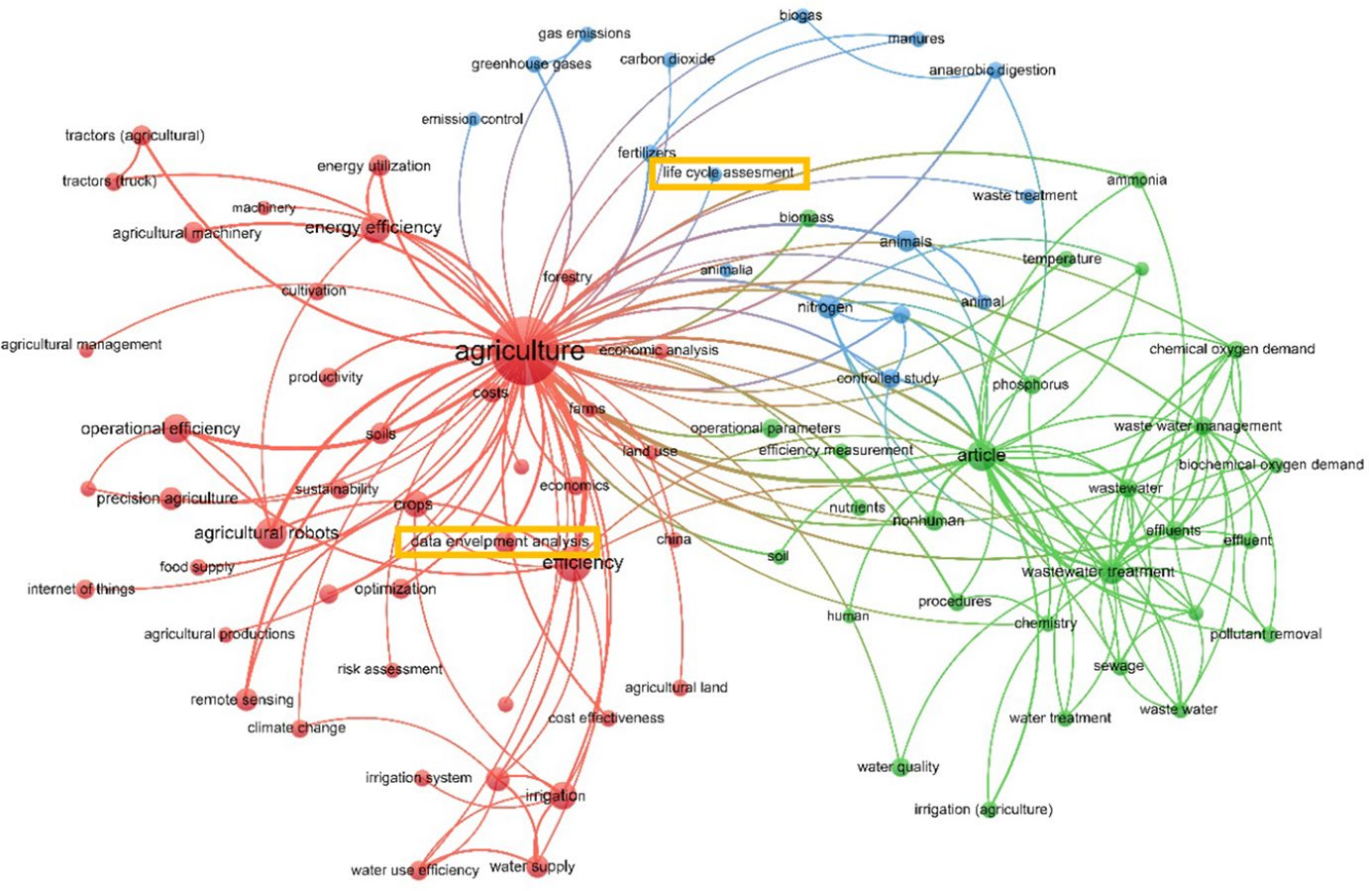
Κeywords’ relationship for agriculture & efficiency terms

In order to assess the way that efficiency measurement is applied in the agricultural sector, VOSViewer software (Waltman and van Ecken 2010) was used. More precisely, Fig. 1 presents efficiency and agriculture results from the most cited papers of Scopus (first 2,000) and Web of Science (WoS) (first 1,000) databases. Three distinct clusters were formed. The first one (red) is referring to operational/technological aspect of agricultural activity, the second (blue) is concerning the environmental impact of either greenhouse gases or agro-chemicals, while the third one (green) is concerning waste water management. DEA and Life Cycle Assessment (LCA) are the only two represented methodologies out of the whole sample. Considering the advantages and disadvantages presented in the previous paragraph as well as the results of Fig. 1, DEA is selected to be further analysed in this literature review.

Focusing on DEA implementation, there are two ways of increasing the overall efficiency of Decision Making Units (DMUs) of the examining system each time, either by reducing the involved inputs (input-oriented) or by increasing the final outputs (output-oriented). Moreover, Constant-Returns-to Scale (CRS) and Variable-Returns-to Scale (VRS) are the most used DEA models, permitting researchers to calculate scale efficiencies as well.

Apart from the conventional DEA models, slack-based models (SBM) can compute further reductions or surpluses, after the initial optimization process. More precisely, slacks are described as technical efficiency remainings, meaning that after the first stage of efficiency computations, further decreases for some variables can be implemented not horizontally, but on a DMU basis. Application of different weights between inputs and outputs is feasible by using assurance region models (Thompson et al. 1996), leading to a fairer benchmarking.

Additionally, newer approaches such as super efficiency models are excluding the examining DMU each time from the reference set, acting as a sensitivity analysis for DEA models (Seiford and Zhu 1999; Thrall 1996). Another model is Network DEA, which can perform efficiency evaluation in different stages of a production process, rather than considering only the initial inputs and final outputs. For instance, production and distribution are two main processes until the products will reach to final stores. By using Network DEA it is possible to optimize the procedure in each stage, without considering the whole system as a black box (Färe et al. 2007; Sarkhosh-Sara et al. 2020). Bootstrap DEA can create replicate datasets in order to check the standard error of their final outcomes (Bogetoft and Otto 2011), a meaningful technique for agriculture which deals with high variability of the involved factors or small samples (Tetteh Anang et al. 2020). Fuzzy DEA model is another approach where the integrated values are not constant, but they are varying within a range, quantifying the risk of the final decisions. Hatami-Marbini et al. (2011) in their literature review paper are presenting different approaches on how imprecise data can be handled under fuzzy concept, while Houshyar et al. (2012) have performed a Fuzzy DEA model so as to assess the sustainability performance of corn farmers. Lastly, Window DEA can be used for measuring efficiency through the use of time-series data. For instance, Pishgar-Komleh et al. (2021) assessed the eco-efficiency of the agricultural sector of European countries for 2008–2017 time period by using the Window DEA method. It should be stated that all the afore-mentioned approaches can handle undesirable outputs (e.g. greenhouse gas emissions) when estimating efficiency scores (Halkos and Petrou 2019), a significant characteristic for considering negative externalities to the environment or human health in the optimization process. Taking all the above mentioned into consideration, this study seeks to address the ways that DEA methodologies are implemented under the prism of sustainability in the agricultural sector.

The study proceeds as follows. Section 2 provides an overview of similar literature reviews in the energy and agricultural sector, clarifying the contribution of this paper. Section 3 presents the overall process of paper collection and screening. Section 4 presents general information of the included papers; DEA model implementation; DEA extensions; Data types used in DEA model; Data collection and processing and sustainability dimensions represented through DEA implementation. Section 5 provides further insights into the acquired results, proposing possible combinations with already existing papers, while in Sect. 6 proposals for future surveys are being made.

## State of the art

The literature review of Zhou et al. (2018) is a crucial reference point, regarding DEA implementation under the sustainability term, indicating the chronological connection of published papers and the key points of DEA evolution from 1996 to 2016, reviewing 320 publications in total. The main conclusions of this study can be summarized as followed (1) Integration of undesirable or bad output in DEA, (2) Interaction of all three aspects of sustainability and the lack of social factor inclusion, (3) Results in adoption from enterprises and policymakers. Another literature review of Mardani et al. (2018), having reviewed 145 articles on the environmental and energy field, concludes that there is a need for further assessment of methodological aspects relative to DEA. Big data, uncertainty, and heterogeneity of the involved DMUs are the main areas that DEA methodology should be further expanded to deal with the complex environment of the energy sector (T. Xu et al. 2020). Tsaples and Papathanasiou (2021) underline also the need for social inclusion, when performing DEA for sustainability. Moreover, on the same survey, it is highlighted that there is a misconception between eco-efficiency and sustainability term, while some authors use more dimensions, apart from economic, environmental, and social, like innovativeness or technology adoption.

The above-mentioned surveys have assessed DEA implementation in Energy and Environmental sectors in total. Considering the idiosyncrasies of the agricultural sector, due to the interaction of multiple factors such as biotic and abiotic environment, cultivation protocols, and applied agricultural practices, including the incorporation of sustainability principles, a literature review of 120 papers was conducted, considering the year after SDGs’ release as a reference point for further promoting sustainability principles in the agricultural operational research society. Although Streimikis and Saraji (2021) have recently published a literature review for DEA in agriculture, focusing on the research gaps and main conclusions of each survey of undesirable outputs, the present review aims to contribute on the following questions:

(1) What are the methodological gaps and the future research proposals?

(2) How are the data collected and analyzed?

(3) What are the methodologies combined or compared with DEA results?

(4) Which of the three pillars of sustainability are covered through published papers or conference proceedings for application of DEA in agriculture?

## Material and methods

**Fig. 2:**
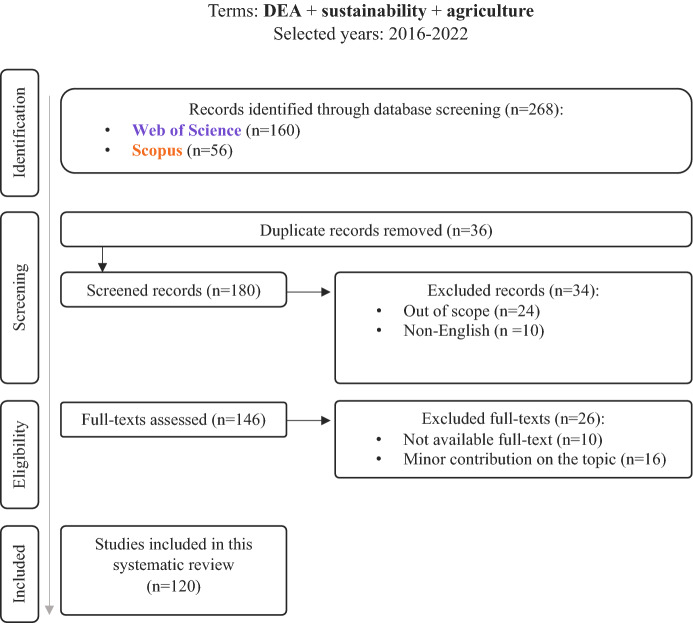
Literature review methodology under PRISMA guidelines

**Fig. 3:**
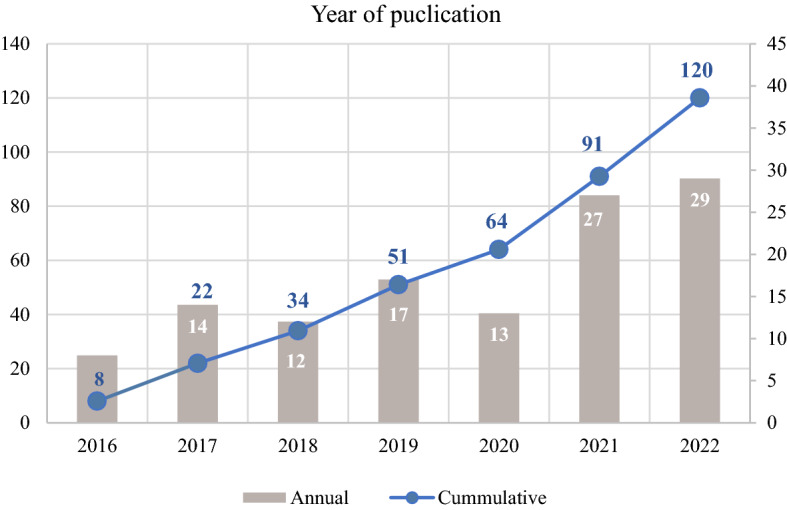
Year of publication

To achieve the aim of this paper, a systematic literature review has been performed through the Scopus and Web of Science (WoS) database, using PRISMA guidelines (Page et al. 2021). More precisely, for this survey terms of «efficiency», «agriculture» and «sustainability» were used. Title, summary, or keywords were the main areas in which the above terms should be present to be included in this research. Due to a large number of acquired results (*n* = 6,960−Scopus and *n* = 7,237−WoS) and the fact that this paper focuses on DEA implementation, the «efficiency» term was replaced with «DEA» term, leading to 75 results from Scopus and 203 from WoS. Given the fact that this literature review assesses the ways in which DEA is applied in agriculture, under the prism of sustainability, a term which was highly promoted after the SDGs’ release in 2015 (United Nations 2015). Having this as a reference point the period 2016–2022 was selected to be further analysed, leading to a number of 180 unique articles or conference proceedings (Fig. 2). Significant academic efforts prior to the selected years have been made in this field (Gerdessen and Pascucci 2013; Reig-Martínez et al. 2011; Zahm et al. 2008), thus this review seeks to capture the contribution of agricultural operational research to sustainability aspects after the year 2016, where there was a rapid increase of publications as Fig. 3 presents.

References were exported on September 9th, 2022. Further screening was performed for removing duplicates and clarifying the content of the included papers. Out of 180 unique records, 34 of them were removed from the first stage due to the fact that 24 paper were not relevant to this literature review (mainly because they were referring in their abstract to the term «agriculture» as a part of an example or as a future implementation) and 10 of them were non-English papers. On the eligibility phase, 26 papers were excluded, 10 of them due to unavailability of full text and 16 of them due to minor contribution on the topic, meaning that in most cases agricultural sector was compared with other sectors mostly in national level but without deepening on agriculture. Based on the above-mentioned process, 120 papers were included in this systematic literature review. Out of the entire set of examined for this review, 116 were journal articles, accounting for 97% of the total, while the remaining 3% were conference papers.

Moreover, a detailed table of criteria, prior to the detailed review of each paper, was constructed based on the authors’ experience in the field. As shown in Table 1, 23 variables were evaluated in each paper. More specifically, the selection of the variables was made to capture the overall picture of the DEA applicability in agriculture under the prism of sustainability, but also to highlight the points that need further amelioration, or better integration of new methodologies from other scientific fields.

**Table 1:** Examined data through the literature review process

Category	Νο.	Element	Description
1. General information	1	Author(s)	(−)
	2	Year	Year of publication
	3	Level	Application-level: International, National, Prefecture, Local
	4	Document Type	Journal article, Conference paper, Proceedings, etc
	5	Source Type	Journal name
	6	Inputs	Type of variables used as inputs
	7	Outputs	Type of variables used as outputs
	8	Application system	Description of application system
2. DEA Implementation	9	Approach	Input-oriented, Output-oriented
	10	DEA Model	Used DEA Models (VRS, CRS, SBM, Window etc.)
	11	Undesirable Output	Use and Type of undesirable output
	12	Homogeneity/Weights	If all DMUs have been treated as homogenous
3. DEA Extensions	13	Combination	Use and Type of any combined methodology with DEA
	14	Comparison	Use and Type of any methodology used to compare DEA results
4. Data Type	15	Qualitative data	Use of qualitative data
	16	Timeseries	Use of data for a longer period than one year
	17	Geographic information system (GIS) Incorporation	Incorporation of GIS information in DEA model
5. Data collection and processing	18	Source of Dataset	Personal interviews, Pubic or Private Datasets,
	19	Total Sample	Number of DMUs
	20	Sample equation	Followed methodology for defining sample size
	21	Software	Which software has been used for DEA implementation
6. Sustainability Dimensions	22	Sustainability Dimensions (in the DEA process)	Which of the 3 aspects of sustainability are assessed in the DEA model
	23	Sustainability Dimensions (in the total paper)	Which of the 3 aspects of sustainability are assessed in the whole paper’s contribution

The above-mentioned data provide further insights on the given dataset of references, leading to the fulfillment of the goals set in the State-of-the-Art section.

## Results

All categories of variables listed in Table 1 are presented in the same order in this section

### General information

Regarding publication year, Fig. 3 presents that there is a noteworthy increase from 2016 to 2022. Apart from year 2020, which was the first year of COVID-19 pandemic, there is an additional amount of publications each year leading to an almost quadrupling of annual publications between 2016 and 2022, signifying there is a great deal of academic interest in this topic.

Table 2 contains the number of reviewed papers by source type, referring to 65 out of 120 papers (54%). *Sustainability*, *Journal of Cleaner Production*, *Science of the Total Environment and Agriculture* were the sources out of which the most papers were extracted for this review.

**Table 2:** Number of included papers by source type

No.	Source type	Number of included papers
1	Sustainability	29
2	Journal of Cleaner Production	9
3	Science of the Total Environment	4
4	Agriculture	4
5	Energies	3
6	Agricultural systems	3
7	Land Use Policy	3
8	Environmental Science and Pollution Research	2
9	Energy	2
10	Information Processing in Agriculture	2
11	Applied Energy	2
12	Energy for Sustainable Development	2

**Fig. 4:**
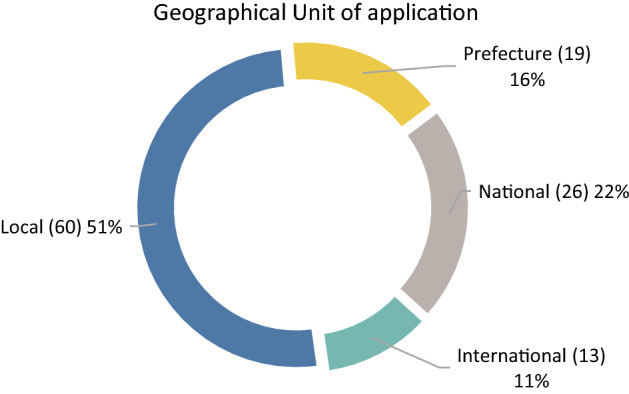
Geographical unit of application

Due to the fact that DEA considers all the involved DMUs as homogenous, it was important to focus more on the geographical aspect of these applications. It is assumed that increased locality of application fits better to the characteristics of the model, mitigating the influence of different external factors. Figure 4 presents that the greatest part of papers (51%) is performed on a local, or regional level. Local level refers to surveys held inside the boundaries of a prefecture, prefecture label refers to the implementation of the survey between neighboring prefectures, national label refers to the inclusion of the majority of prefecture inside a country and lastly, international label refers to the comparison of agricultural sectors between different countries. It should be noted that in this figure 118 papers are included, because the remaining two are review papers.

**Fig. 5:**
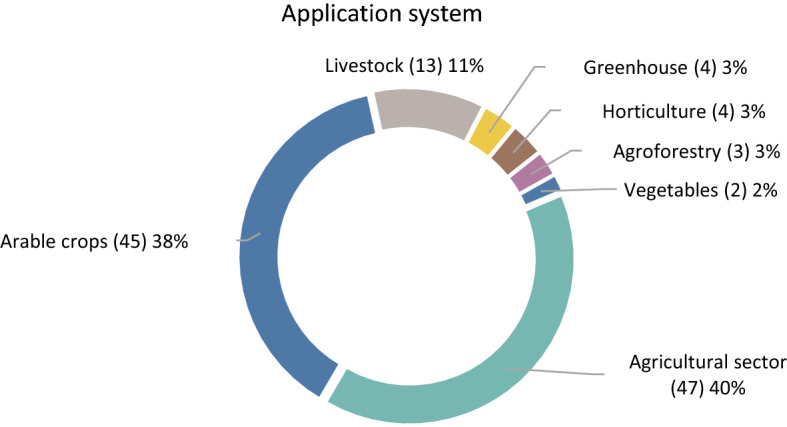
Application system

Regarding the application system, Fig. 5 presents that a great part of the examined papers are referring to the agricultural sector in general, 38% implements optimization models for arable crops and a small part is referring to livestock, greenhouse products, fruits, timber and vegetables.

Table 3 (Appendix section) provides an overall view of included papers in this review. More particularly, author, year, application level, type of inputs and outputs as well as the specification of the application are presented.

### DEA implemantation

Regarding the selected approach, 76% used an input-oriented approach, 20% used an output-oriented approach, 2% compared the approaches of both results, and 2% did not specify the approach used.

**Fig. 6:**
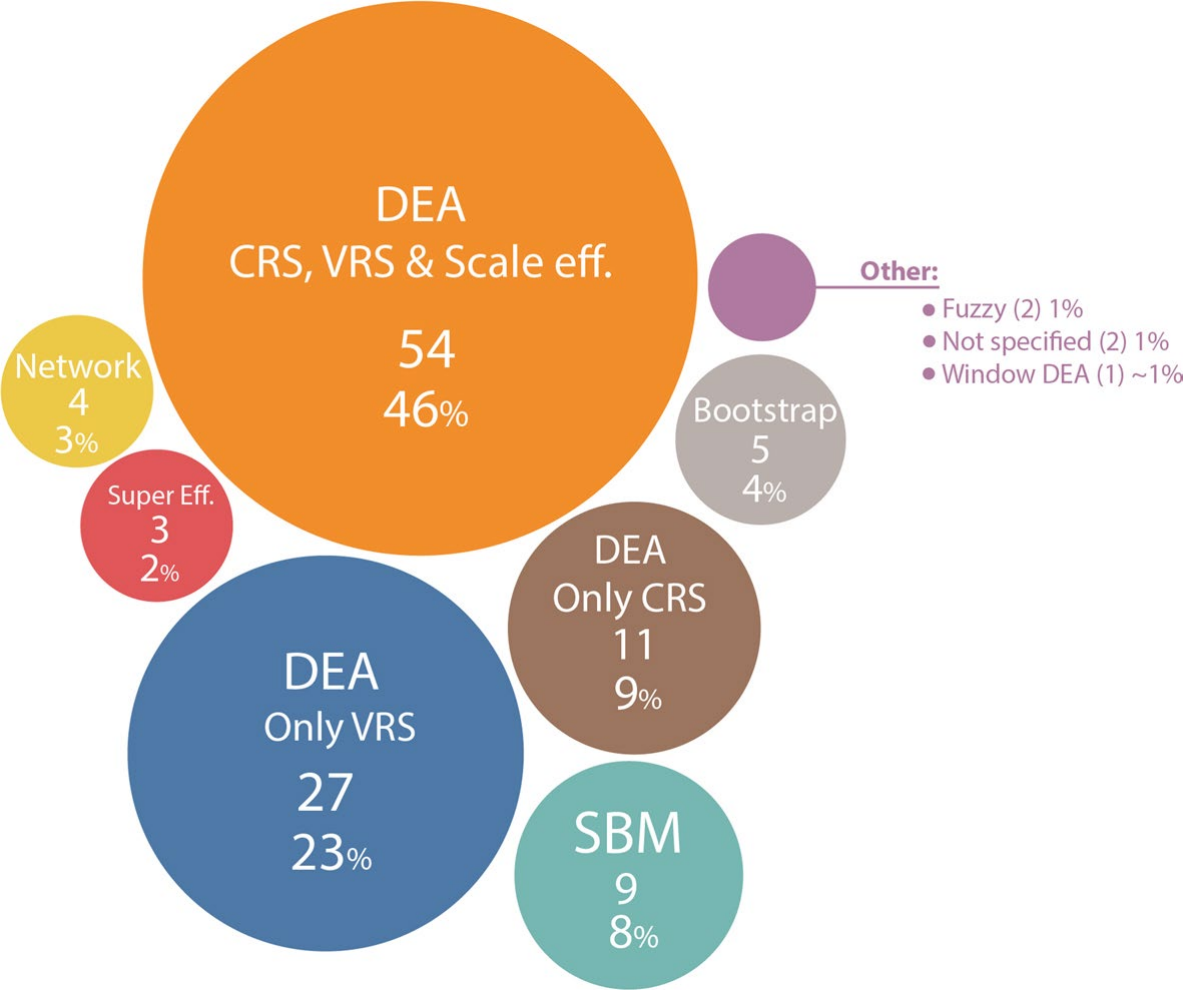
Results of DEA models used

Examining the use of DEA models in agriculture, it is evident that most of the obtained results were acquired using typical DEA models like CCR (CRS) and BCC (VRS). Particularly, as shown in Fig. 6, almost half of the examined papers (46%) are using both CRS, VRS and Scale efficiency, 23% used only VRS model (27 papers) and 9% used only CRS model (11 papers. Although the selection of the CRS or VRS approach is problem specific, in agricultural sector VRS assumption is preferred, due to the fact that the increase of inputs does not mean necessarily that this will lead to a proportional increase of outputs. In other words, doubling inputs (e.g. fertilizer) does not ensure double production in the end of the cultivation year. CRS scores are mainly extracted for scale efficiency calculations.

Additionally, Slack-based model (SBM) was used from 9 papers. As mentioned in the Introduction section, SBM models are used to provide accurate estimations of target values of each variable enabled in the DEA model. Debbarma et al. (2021) used SBM model to elucidate Iranian farmers' efficiency under the consideration of GHG emissions as undesirable output, while same model was used from Tian et al. (2016) for open-field grape production. Bootstrap DEA was used in 5 cases with a view to minimize the stochastic errors by producing replicate datasets. For instance, Nodin et al. (2022) have created 3,000 replicate datasets of rice producers to assure the reliability of acquired results. Super efficiency was performed by 3 papers or 2% of total sample. Cecchini et al. (2021) used this approach for minimizing the influence of extreme values to their final results when implementing an efficiency assessment on Italian sheep farms.

Network DEA was also implemented from 5 papers in order to reveal causes of inefficiency in different sub-systems of an overall process. Saputri et al. (2019) performed this methodology to assess the efficiency between the three distinct stages of agri-food supply chain (agricultural production, processing, transportation) for Indonesian rice producers. Kord et al. (2022) presented agricultural activity as two different stages (environmental and economic) and by using shared inputs between the two stages they performed a sustainability assessment for Iranian regions. Lu et al. (2022) have created a three stage Network model for assessing agricultural food production systems of EU countries under circular economy principles, meaning that the final output was acting as a carry over the next period.

Fuzzy DEA and Window DEA were the least presented methodologies of this sample referring to only 3% cumulatively. Mu et al. (2018) have assessed 55 dairy farms setting a range of −20 to + 20 of their given values, so as to incorporate the uncertainty in their Fuzzy DEA model. Window DEA was used from Masuda (2019) to minimize the effects of global warming and eutrophication in rice production for 2005–2011 time period. Lastly, it should be mentioned that the followed methodology was not specified in 2 papers and the review papers (2) are excluded from this review process.

Regarding the comparison of the acquired results, only two surveys have proceeded to this step. W Kamal and Ilmas (2017) have compared their DEA results with SFA concluding that SFA technical efficiency results were higher than the ones of DEA, attributing this to bias correction of the SFA model. Khanjarpanah et al. (2017) implemented 2 types of cross-efficiency DEA models (aggressive and benevolent) to assess switchgrass cultivation in Iran and they proposed a third one additional model which contributes to a fairer optimization process.

Undesirable outputs impact assessment is another significant factor towards the achievement of sustainable development in agriculture, mainly by focusing on reducing their impacts on the environment, or trying to create a circular path. For these reasons, 25% (30 papers) used undesirable outputs in total. Most of them were using either Greenhouse Gas Emissions (GHG) as a total or applying CO_2_ emissions only and this may be due to easy data accessibility. Lamkowsky et al. (2021) has also used N surplus indicator as undesirable output in Dutch dairy farms, a variable which has not been detected in crop production systems at all (e.g. N leaching). Additionally, Tang et al. (2022) included farm-specific undesirable variables such as soil erosion rate and grey water footprint in their DEA model, a characteristic that was absent from the other surveys. It should be noted that Grassauer et al. (2021) and Rybaczewska-Błazejowska and Gierulski (2018) included LCA results as inputs in their DEA models in order to minimize the environmental effects of agricultural productivity.

As stated in the general information section, there is an assumption of homogenous examined units when performing DEA. Especially in the agricultural sector, which has a great variability both of abiotic (temperature, humidity, precipitation, type of soil etc.) and biotic environment (cultivar, variety, pests etc.) as well as the interaction between them, use of different weights is essential for setting an equal starting point for all DMUs involved. None of the included references has implemented any methodology that would make a fairer evaluation, a crucial point when considering equality on the agricultural sector. Such issue is partially delivered from Molinos-Senante et al. (2016) where an attempt of highlighting efficiency differences between farmers, with immediate access to water or not is being made, underlying the need for policy framework modifications. In this line of reasoning, other agronomic factors such as access to land with high levels of organic matter, or vulnerability from specific pests should be considered in the evaluation process.

### DEA extensions

DEA has not been combined with any other model or methodology for 30% of the examined references, proving that most researchers are implementing additional steps after the calculation efficiency scores. From the remaining 82 papers, regression models was the most frequent option such Tobit (10), Truncated (4), Ordinary Least Squares (OLS) (4) and other not specified linear regression models (6). Tobit model was used for checking which socio-economic variables are affecting the extracted efficiency scores (Hassen et al. 2017; W Kamal and Ilmas 2017). As mentioned from (Chang et al. 2022) the use of OLS model can be biased due to the potential inclusion of zero values extracted from the DEA implementation process. Martinsson & Hansson (2021) have used OLS to assess the effect of subsidies in the performance of dairy farms and their overall productivity. Frangu et al. (2018) incorporated in their linear regression model aspects like farmers training on crop nutrition or type of power source used (e.g. electricity, fuel) fulfilling also other dimensions than the typical social characteristics (e.g. age, education, income, years of experience).

Apart from the regression models Malmquist index was used in 10 cases in order to check efficiency differences between years. Pan et al. (2021) used Malmquist index to assess differences of total factor productivity between the years 2015–2018, proving that there was a significant increase in productivity of various Chinese regions. Ren et al. (2017) appied the same index to depict the water use efficiency per year in order to propose regional changes to policymakers. Another least explored index used in combination with the DEA is Theil index, which was used for exploring economic inequalities between different Chinese regions regarding their eco-efficiency (Pang et al. 2016).

LCA is another commonly combined analysis with the DEA for assessing the environmental impacts of agricultural activities. In the examined sample, 14 papers (11%) implemented the afore-mentioned methodology. When LCA is applied there are two approaches of either implementing DEA in the initial stage and then target values are used (Grados et al. 2017), or LCA is performed first and its results are proceeding to further analysis with the DEA (Rybaczewska-Błazejowska and Gierulski 2018). For instance, Mohammadi et al. (2022) have assessed the impacts of agricultural activity to air, water and soil, clarifying the differences between current and target values for Iranian wheat farms.

Principal component Analysis (PCA) and Factor Analysis (FA) were used from a small number of papers (4). After the collection of economic, social and environmental data, Sánchez-Zamora and Gallardo-Cobos (2019) have applied PCA for grouping Spanish regions with common characteristics to measure and compare their resilience scores, extracted from DEA. Ramos de Oliveira et al. (2022) implemented PCA in order to elucidate the interactions among the sustainability factors, proving that social and environmental dimensions should not be neglected when transportation routes of agricultural products are being assessed for their efficiency levels.

Kord et al. (2021) have incorporated a sensitivity analysis in their approach, to assess the allocation of human resources in a 2 stage Network DEA model. More precisely, this paper seeks to address the optimal value of human resources intervention in the plantation/maintenance of the cultivar (first stage) and harvesting (second stage). Abbas et al. (2022) used the aforementioned analysis, so as to indicate the change of crop output under the condition of different number of inputs each time. Grey relational analysis was applied to check the influence of the included variables to the environmental performance of China’s families (Y. Yang et al. 2019).

Lastly, special attention was paid to the incorporation of spatial characteristics in the reviewed papers. Tian et al. (2016) have implemented spatial analysis after estimating the efficiency scores for Chinese grape farms. Spatial Durbin Model was impemented from the following researchers to identify technological spillovers through different regions (J. Li et al. 2021; Wu et al. 2022; P. Xu et al. 2022). Examining the spatial relationship of the acquired results is a necessity for agricultural operational research to reveal potential patterns that may have been neglected in the analysis process.

### Data type

A similar pattern of used inputs Pesticides, Diesel, Electricity, Fertilizers, Labor, Machinery, Seeds, and Yield as used output is revealed through this process. However, it is should be underlined that irrigation has been used only from 24 surveys, raising awareness about data collection and data availability of such a valuable natural source. As it was also mentioned in the data analysis section, farm data regarding agronomic characteristics are missing. This situation does not permit researchers to perform a fairer assessment, treating all the involved DMUs as homogenous.

Apart from the quantitative variables, none of the papers used qualitative variables (e.g. Likert scale) when performing DEA, a valuable characteristic for assessing agronomic characteristics which cannot be easily or precisely measured or quantified. Cook (2004) provides the appropriate methodology on how the incorporation of qualitative data can be implemented. Considering time-series data, 34 out of 118 included references have analyzed data of more than one year. Authors selected to include this variable in order to check the validity of acquired results that may present high variations due to external factors. For instance, bad weather conditions can result in small yield for one region, perceiving it as inefficient compared with another one in the same year. Seasonal differences should be carefully considered when DEA is applied in agriculture. It should be also highlighted that only one survey has applied Window DEA to treat time-series data (Gatimbu et al. 2020), which is the most appropriate methodology for this type of data. Moreover, only none of the studies has incorporated any information from GIS system, highlighting the need for acquiring up-to-date data in an easier and more precise way. In this way, farms or regions can be better characterized, setting on the optimisation process all their unique features that may influence the validity of acquired results.

### Data collection and processing

Although there is a detailed record of all the included sources, in this review four larger groups were created. Data were collected through; public databases (EUROSTAT, FADN, FAOSTAT, China Statistical Yearbook, other sources) by 49% (58 papers); personal interviews by 45% (53 papers) funded project collaboration by 3% (3 papers); private sector by 3% (2 papers) and not specified in one of them. It should be mentioned that Seo and Umeda (2021) used data from field experiments, an aspect which was absent from this literature review process and should be further promoted for acquiring accurate results. Total sample size has been added as a variable to check the rule of thumb for the ratio of DMUs involved compared to the number of examined variables. None of the examined papers appeared to be problematic on that.

Focusing on data collection through personal interviews, a small part of them (12 papers) had a reference on how they collected their samples. More precisely, 4 referred to Random sampling technique formula (Raheli et al. 2017; Ramezani et al. 2022; Sherzod et al. 2018; Sui et al. 2022); another 4 to Cochran technique (Ashraf et al. 2020; Esfahani et al. 2017; Molinos-Senante et al. 2016; Payandeh et al. 2021); 2 to Yamane technique (Haq and Boz 2019; Ul Haq et al. 2020); 1 to Stratified Sampling formula (Godoy-Durán et al. 2017) and one to snowball sampling method (Mwambo et al. 2021).

**Fig. 7:**
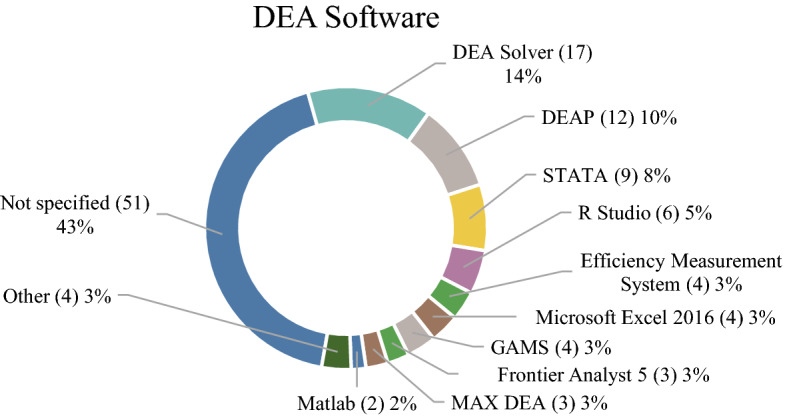
DEA software

To authors’ surprise, the greatest part of the papers (51%) did not specify which DEA software they used to acquire DEA results, which would be helpful for results reproducibility. DEA Solver, DEAP, and STATA were the most used as shown in Fig. 7. Regarding the RStudio software, Benchmarking library was used in 4 papers while deaR library in another 2.

### Sustainability dimension

**Fig. 8:**
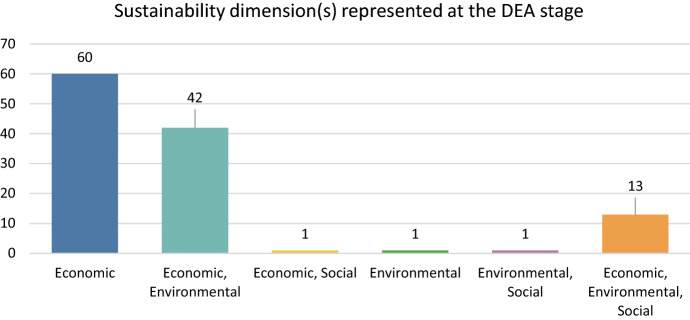
Sustainability dimension(s) represented at the DEA stage

Lastly, all papers were grouped by the sustainability dimension that they represent. Although there is a large discussion about how we can define sustainability and which aspects should be included (Purvis et al. 2019), for the scope of this review sustainability is represented by the three aspects of economic development, environmental protection, and social inclusion. It should be mentioned that categorization was made based on two stages. The first stage was referring to the variables inserted immediately in the DEA model, while the second stage was examining the overall contribution to sustainability assessment. For instance, if a paper was using typical inputs and outputs (e.g. labour, fertilizers, land, energy and overall production), it was perceived as solely economic. When a paper has included in the above stated variables an undesirable output (e.g. GHG emissions) or LCA results, it was classified in the economic and environmental category. There were also 2 cases in which Human Development Index (HDI) (Babazadeh et al. 2018; Khanjarpanah et al. 2017) was used in the optimization process, meaning that at the DEA stage the social aspect was represented. As Fig. 8 shows, at the DEA stage half of the papers are contributing only to the economic aspect, 35% concerns both economic and environmental aspect, while only in 11% of the examined papers are representing all sustainability dimensions. For instance, Tang et al. (2022) have incorporated land cost, HDI, annual precipitation and amount of water resources covering all three aspects of sustainability. Sánchez-Zamora & Gallardo-Cobos (2020) have embodied 22 indicators covering economic, environmental, social, institutional and spatial development characteristics.

**Fig. 9:**
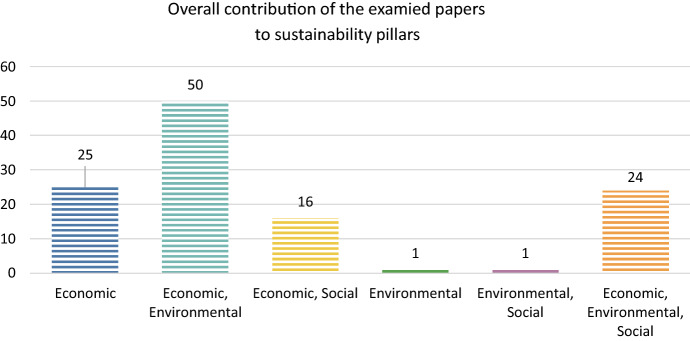
Overall contribution of examined papers to sustainability pillars

Following the same rationale as at the first stage, the examined papers were categorised by the total combination of variables and methods that they implemented in their approaches of sustainability. Figure 9 shows that there is a shift from solely *economic* perspective of Fig. 8 to a combined *economic and environmental *approach. In other words, in many cases where only economic pillar was represented in a DEA methodological approach, authors embodied methodologies such as LCA (Beltrán-Esteve et al. 2017; Gamboa et al. 2020) or functions for the calculation of CO_2_ emissions (Ashraf et al. 2020; Basavalingaiah et al. 2020; Ilahi et al. 2019) or environmental cost benefit analysis (Mwambo et al. 2020). Economic and social aspect increased as well, due to the fact that DEA outcomes were used as dependent variables in regression models such as Tobit (Haq and Boz 2019; Sherzod et al. 2018) or truncated regression (Liu and Sun 2019; Martino et al. 2016), to identify significant relations of socioeconomic variables to them. It is really positive the fact that the number of DEA papers contributing to all sustainability pillars increased from 13 to 24, representing almost 20% of the sample, thus the percentage remains low given the fact that examined papers have been retrieved through a structured search for sustainability in agriculture.

**Fig. 10:**
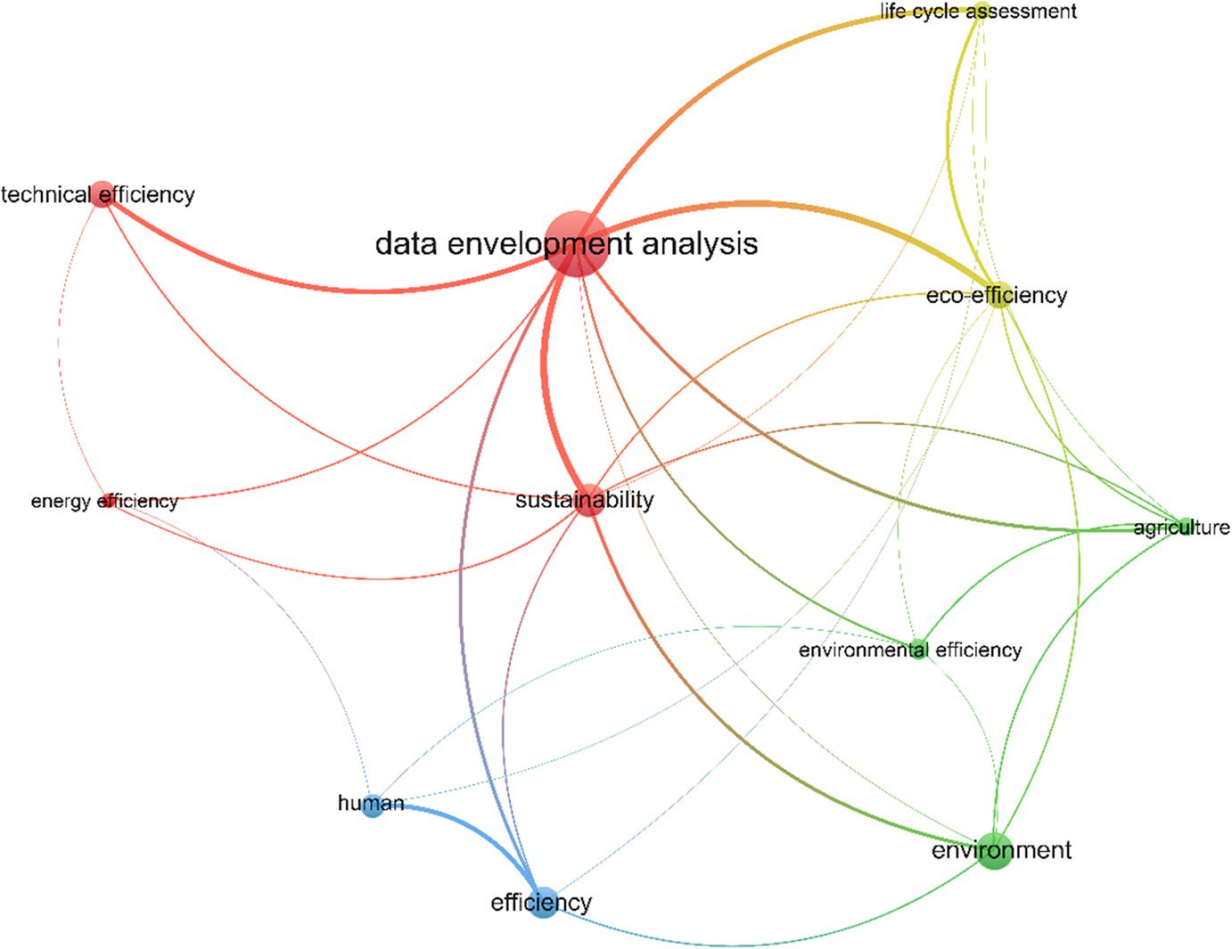
Keywords’ relationship of the included papers

As a final part of this review, obtained results were visualized to provide a clear image to the reader. As expected “sustainability” term is closely related to DEA. LCA term is also present, meaning that authors either refer to the applicability of this method in their papers, or they implement it in combination with DEA, a result which was extracted from Sect. 4.3. In the lower left corner of Fig. 10, there is the label “human” which indicates that even if the number of documents including social features remains small, this term is under authors’ consideration.

## Discussion

The main objective of this study is to identify methodological gaps and propose future directions for the operational research field of agriculture, considering sustainability as a driving force. Additionally, the contribution of the reviewed papers to the fulfillment of the 3 aspects of sustainable development was evaluated. The significance of this literature review does not stem from its findings but from highlighting missing aspects or points that needs to be improved.

Over the years there is a clear approach of constantly finding new methodologies to better integrate the concepts of reduced resource availability and environmental protection in DEA methodology. As Galanopoulos et al. (2006) stated, farmers can only control their inputs and they have less impact on the final output, due to a series of external factors. This is the reason why the input-oriented approach is selected, to minimize the risk of the invested capital from the farmer’s side as well as promote environmental protection through reduced use of agrochemicals. Although fertilizers skyrocketed the production potential on a global scale, high amounts of energy are needed for their production and distribution (Dimitrijević et al. 2020). This is another reason why the input-oriented approach is selected, leading to production systems with lower energy requirements.

Results indicate that the greatest part of surveys was held out on a local level, thus DEA remains a handful tool for measuring the performance on a greater scale. However, none of the examined papers have assessed the infrastructure of agricultural domains for each country. For example, how the funds of EU agricultural sectors are distributed in subsections like crop production, livestock production and mixed systems, through hierarchical network models (Kremantzis et al. 2022). It should be also underlined that the implementation of weights would lead to more reliable results (Mosbah et al. 2020; Thompson et al. 1994, 1995). This is a point of great importance for the agricultural field, where multiple external factors affect the interactions of the used inputs, also influencing the final output. For example, temperature affects nitrogen release rates depending on fertilizer type or soil type (Ransom et al. 2020), soil pH plays an important role in plant growth (Xiao et al. 2017), salinity (Hessini et al. 2019), and a series of factors that affect the final output can be inserted in DEA model as weights. That is the reason why the incorporation of GIS information in DEA methodology is essential, but there is a limited number of papers available online with this combination (Liang et al. 2019).

Estimation of undesirable outputs is another point of interest for agricultural productivity. Literature review shows that most researchers use CO_2_ or GHG emissions to align their papers with the global effort for GHG emissions reduction. These outcomes are in accordance with Streimikis and Saraji’s (2021) review results. However, there is an increasing need for creating circular flows to eliminate the wasted energy, supporting this transition by an appropriate policy framework (Guo et al. 2021).

Moreover, it should be stated that data availability remains an issue in the agricultural field. Almost half of the surveys used data acquired through personal interviews, proving that data collection is a time-demanding process that also involves an increased risk of imprecise data. On top of that, researchers have limited access only to basic information as shown in Table 3, mainly because additional data collection requires an establishment of greater infrastructure e.g. agro-related applications where farmers insert either manually or automatically their data, local agro-managers provide a first stage data screening and lastly, researchers provide further insights and results from visualization. Further assessment is needed regarding qualitative data like the quality of sowing, quality of spraying or quality characteristics of the final product. TOPSIS Model, which can handle both scale and categorical data, can be easily combined with DEA methodology in the agricultural sector, embodying a wider range of involved variables in the benchmarking process (Kyrgiakos et al. 2021a, b; Wang et al. 2021).

Additionally, out of the 34 papers that used time-series data, 31 extracted them from public datasets, 1 from project collaboration, and another 1 from the private sector. By this statement a lack of constant monitoring by cultivar type and by specific region is highlighted, as the remaining papers performed an annual analysis, indicating the need for incorporating a greater part of the variability, derived from of multiple years analyses.

The social dimension is the least represented aspect when measuring efficiency in agriculture under the sustainability framework, a conclusion that derives both from the present review, but also has been highlighted in literature reviews of energy and environmental fields (Tsaples & Papathanasiou 2021; Zhou et al. 2018). Moreover, even though the economic dimension is the most highlighted one in Fig. 8, it should be considered that when estimating the potential reduction of the amount of fertilizer per land unit, it is apparent that this act enhances environmental protection. Though the main outcome of this survey, that the social aspect is still underrepresented, as highlighted in Fig. 9.

The limitation of this research lies in the fact that the sources were extracted only by using a firm approach of paper selection, searching for DEA and sustainability and agriculture «terms» on their title, abstract, or keywords. Although authors are aware of the existence of a higher number of papers with DEA implementation in the agricultural sector with great potential, e.g. application of DEA in agriculture at the EU level (Kočišová 2015; Madau et al. 2017), local level (Işgın et al. 2020), comparisons of DEA results with SFA (Theodoridis and Psychoudakis 2008) or newer approaches like 2-stage DEA (F. Ren et al. 2021), engagement of spatial characteristics (Z. Li et al. 2021), DEA with Artificial Neural Networks (ANNs) (Vlontzos and Pardalos 2017) or Window DEA approaches (Kyrgiakos et al. 2021a, b; Shahraki et al. 2019), thus they were excluded because they did not fulfill the previously stated limitation. Moreover, prominent journals like American Journal of Agricultural Economics or Journal of Agricultural Economics are missing from the two databases, a fact that should be seriously considered by researchers when using these search engines.

Eco-efficiency was another serious consideration when designing this survey due to the fact that there is a considerable effort of several researchers under this term as well (Gómez-Limón et al. 2012; Kiani Mavi et al. 2019; Rebolledo-Leiva et al. 2019). However, using this specific term the pillar of environmental protection would be overestimated and this may lead to non-objective results. Taking the above-mentioned limitations into consideration, authors agreed to proceed with this approach, assuming that the sample size is representative and can provide a simple and realistic overview to the reader. As a final remark, the “agriculture” term should be placed in title, abstract, or keywords section from the future authors, to easily distinguish their papers from closely related ones.

## Conclusions

In this literature review, 120 papers were included referring to the use of DEA in the agricultural sector considering sustainability. Results indicate that there is a need for a more systematic data collection that will incorporate data of agricultural practices (both quantitative and categorized), weather data, as well as an effort of combining DEA methodology with information extracted from GIS databases. Also, it is a necessity to perform optimization methods on a multiple-year basis, to engage all the involved variability. Such applications will permit the implementation of more complex DEA models with greater adaptability in real-case scenarios. The integration of weights in DEA models can contribute to achieving the above goal, ensuring the same baseline before the benchmarking process. Additionally, it is necessary to integrate social factors, especially in cases where the aim of the research is to provide information to policymakers. Concluding, data availability and implementation of more complex methodologies are needed to acquire results with greater explanatory power, contributing to the achievement of sustainable development principles in the agricultural sector.

## Data Availability

The data are available upon request to the corresponding author.

## Appendix

See Table 3.

**Table 3:** All the included references of the reviewed papers

Authors	Ap. Lvl	Inputs	Outputs	Application system
Skevas and Serra (2016)	L	Capital, Labor, Land, Other costs, Pesticides, Fertilizers	Production (Euro)	Arable farms
Pang et al. (2016)	N	Agricultural Machinery, Agricultural film input, Fertilizers	Added Value	Agricultural sector
Tian et al. (2016)	N	Agricultural film input, Agro-chemicals, Diesel, Electricity, Irrigation, Labor, Organic Fertilizers	Production	Grapes
Baležentis et al. (2016)	N	Energy, Labor, Real Fixed Capital Stock	Added Value	Agricultural sector
Molinos-Senante et al. (2016)	L	Energy, Labor, Pesticides, Fertilizers	Production	Agricultural sector
Ghali et al. (2016)	L	Capital, Intermediate consumption, Labor, Land	Crop output, Livestock output, Other Output	Agricultural sector
C. N. Wang et al. (2016)	L	Equity, Liabilities, total assets	Gross Profit, Net Revenue	Agroforestry
Martino et al. (2016)	L	Capital, Labor, Land, Other costs	Gross Product	Agricultural sector
Vlontzos et al. (2017)	I	Agro-chemicals, Capital, Energy, Labor, Land	Production (Euro)	Agricultural sector
C. Ren et al. (2017)	L	External hidden flow, Import water resources, Local water resources	Local waste output, Local waste system	Agricultural sector
Grados et al. (2017)	P	Agro-chemicals, Labor, Machinery, Seeds, Fertilizers	Production	Potato
Raheli et al. (2017)	L	Agro-chemicals, Diesel, Irrigation, Labor, Machinery, Manure, Seeds, Fertilizers	Yield	Tomato
Godoy-Durán et al. (2017)	L	Economic, Environmental, Social	Yield	Horticulture
Esfahani et al. (2017)	L	Electricity, Land, Manure, Fertilizers	Yield	Corn
Beltrán-Esteve et al. (2017)	L	Income	Ecotoxicity, Eutrophication potential, Global Warming Potential, Human toxicity, Lentil, Ozone	Citrus
Rebolledo-Leiva et al. (2017)	P	Energy, Land, Machinery, Pesticides, Raw materials, Fertilizers	Packaging residues, Production	Blueberry
Lee and Park (2017)	L	Agro-chemicals, Diesel, Electricity, Irrigation, Labor, Machinery, Seed, Fertilizers	Production, straw	Soybeans
Y. Wang et al. (2017)	P	Agro-chemicals, Irrigation, Machinery, agricultural population, blue water, green water, irrigated area	Production, Production (Euro)	Agricultural sector
Hassen et al. (2017)	L	Agro-chemicals, Ferilizers, Labor, Land, Machinery, Seeds	Production	Wheat
Martinho (2017)	I	Agro-chemicals, Labor, Labor wages, Fertilizers, total assets	Total Output	Countries
Khanjarpanah et al. (2017)	P	Annual percipitation, Human Development Index, Irrigation, Land, Population, percipitation, temprature, unemployment index	Production	Switchgrass
Kamal and Ilmas (2017)	N	Feed, Labor, Medication, Number of animals, Utilities	Production (Euro)	Broiler
Varela-Candamio et al. (2018)	P	Capital, Labor, Land, Shadow price	Production, Subsidies	Agricultural sector
Rybaczewska-Błazejowska & Gierulski (2018)	I	Agricultural Land Occupation, Climate Change, Freshwater Eutrophication, Freshwater ecotoxicity, Human toxicity, Ionizing radiation, Marine Ecotoxicity, Marine Eutrophication, Ozone depletion, Terrestial acidification, Urban Land Occupation, fossil depletion, metal depletion, natural land transformation, photochemical oxidant formation, terrestrial ecotoxicity, water depletion	Gross ouput value	Agricultural sector
Sherzod et al. (2018)	L	Labor, Seeds, Fertilizers	Production	Wheat
He and Zhang (2018)	L	Quality management system, Quality improvement plan, Relative price level per employee, Training time, Equipment Environmental amelioration Cost input rate of research funding	Product qualification rate, Rate of return on total assets, Quick ratio, Profit growth rateon-time delivery, Rate order completion Rate enterprise reputation, Information level, Strategic objective compatibility, carbon dioxide emission, “three wastes” recycling rate	Agricultural sector
Babazadeh et al. (2018)	N	Annual percipitation, Cost of cultivation, Human Development Index, Land, Number of oil extraction, Population, Sunlight, Water resources	Production	Rapeseed, Soybeans
Dong et al. (2018)	P	Area, Labor, Machinery, Nitrogen	Added Value, CO2 emissions	Agricultural sector
Mutyasira (2018)	L	Farm Sustainability Index	Economic, Environmental, Social variables	Agricultural sector
Izadikhah and Khoshroo (2018)	L	Labor, Machinery, Seed, Fertilizers	Total Production	Maize
Mu et al. (2018)	L	Land, Livestock units, Milk costs, fat content, protein content	Energy use, Gross Margin, Land use, N Surplus, P Surplus	Dairy farms
Frangu et al. (2018)	P	Labor, Land, insecticides	Production	Greenhouse Vegetables
Xing et al. (2018)	N	CO2 Emissions, Energy Usage, Exhaust emission, Hazardous Waste Generation, Waste water discharge, Water withdrawl	Economic output	Multiple Production Sectors
Abbas et al. (2018)	L	Chemicals, Diesel, Electricity, Human labor, Irrigation, Machinery, Seed, Fertilizers	Yield	Wheat
Masuda (2019)	L	Agro-chemicals, Buildings, Electricity, Land, Machinery, Production costs, Seeds, Fertilizers	Yield, Yield (Straw)	Rice
Rodrigues et al. (2019)	P	Economic, Environmental, Social	Production (Euro)	Fisheries
Sintori et al. (2019)	L	Feed, Labor, Land, variable capital cost	Meat, Milk	Sheep Farms
Dania et al. (2019)	T.M	Economic, Environmental, Social	System Requirments	Agricultural sector (Supply Chain)
Saputri et al. (2019)	L	Capital, Energy, Labor, Material and Service, Services	Production	GMO & non-GMO Products
Tan et al. (2019)	N	Equity financing cost, Interest expenses, Investment in innovation, Main business cost, Total debt	Economic value added, Return on asset ratio, Weighted average return	Agricultural businesses
Liang et al. (2019)	P	Agro-chemicals, Labor, Land, Manure, Material and Service, Seeds, Fertilizers	Gross ouput value	Greenhouse Vegetables
Bournaris et al. (2019)	P	Agro-chemicals, Area, Labor, Other costs, Seeds, Fertilizers	Gross Returns	Greenhouse Vegetables
Elhag and Boteva (2019)	L	Agro-chemicals, Diesel, Electricity, Irrigation, Labor, Seeds, Fertilizers	Yield	Greenhouse Vegetables
Grados and Schrevens (2019)	L	Labor, Machiner, Ferilizers, Pesticides, Fungicides	Production	Potato
Sánchez-Zamora and Gallardo-Cobos (2019)	P	Economic, Environmental, Social	Composite indicator	Agricultural sector
Ilahi et al. (2019)	P	Chemicals, Diesel, Electricity, Labor, Machinery, Seeds, Water, Fertilizers	Yield (Wheat grain)	Wheat
Ozden and Ozer (2019)	N	Capital, Labor, Land, Pesticides, Population, cattle stock, Fertilizers	Production (Euro)	Agricultural sector
Haq and Boz (2019)	L	Labor, Fertilizers	Production	Tea
Y. Yang et al. (2019)	L	2 models, Ferilizers, Fertilizer per Ha, Labor, Land, Machinery, Pesticide per Ha, Pesticides, Seeds	Crop output	Crop Farms
Liu and Sun (2019)	L	Land, Other costs, Fertilizers	Bamboo roots, Bamboo wood, Production, Production (Euro)	Timber
Watto and Mugera (2019)	L	Agro-chemicals, Farm size, Irrigation, Labor, Seed, Fertilizers	Yield	Wheat
Gatimbu et al. (2020)	L	Capital, Energy, Labor, Land, Material and Service	Only undesirable outputs	Tea
Gamboa et al. (2020)	L	Labor, Machinery, Pesticides, Fertilizers	Production	Quinoa
Coluccia et al. (2020)	N	Gross Capital, Irrigation, Labor, Land, Fertilizers	Production	Agricultural sector
(Ul Haq et al. 2020)	L	Labor, Fertilizers	Production	Tea
Pereira Domingues Martinho (2020)	I	Energy costs, Fixed assets, Labor	Total Output	Agricultural sector
Mwambo et al. (2020)	L	Agricultural Services, Evapotranspiration, Human labor, Seeds, animal Labor, Fertilizers, topsoil loss	Yield	Wheat
Ayouba and Vigeant (2020)	L	Capital, Labor, Land, Pesticides, Raw materials	Production	Wheat
Bartova and Fandel (2020)	L	Energy, Fixed assets, Labor, Land	Production	Wheat
Ashraf et al. (2020)	L	Agro-chemicals, Diesel, Electricity, Irrigation, Labor, Machinery, Seed	Yield (Cereals), Yield (Straw)	Wheat
Basavalingaiah et al. (2020)	L	Agro-chemicals, Diesel, Energy, Farmyard manure, Irrigation, Labor, Machinery, Seeds, Fertilizers	Grain Yield, Yield (Straw)	Rice
Nguyen et al. (2020)	L	Crop production cost, Labor, Land, Other costs, Pesticides	Production	Orange
García-Cornejo et al. (2020)	L	Depreciation expenses, Labor, fixed capital cost, variable capital cost	Production (Euro)	Dairy farms
Nguyen et al. (2020)	N	Land, Family labor, Outsourced labor cost, Crop Production cost, Other expenditures	Production	Oranges
Grassauer et al. (2021)	N	Aquatic ecotoxixity, Cumulative energy demand, Global warming potential, Normalized eutrophication	FNI, HNVf, net food production-protein	Agricultural sector
Lucas et al. (2021)	I	Acidification, Eutrophication, Fresh water withdrawls, GHG emissions, Land Use	Calories, Qualifying index	food sector
Domagała (2021)	I	Energy, Labor, Land, Fertilizers	Production, net value added	Agricultural sector
Papadopoulou et al. (2021)	L	Labor, Number of animals, fixed capital cost, variable capital cost	Net income	Small ruminant farms
Kord et al. (2021)	P	Area, Irrigation, Labor	Production, Production (Euro)	Agricultural sector
Khoshroo et al. (2021)	N	Biocides, Electricity, Energy, Machinery, Fertilizers	Production (Euro)	Tomato
G. Singh et al. (2021)	L	Agro-chemicals, Biocides, Electricity, Fuel, Irrigation, Labor, Machinery, Seeds	Grain Yield	Wheat
Cecchini et al. (2021)	L	Area, Feed, Labor, Livestock units	Meat, wool	Sheep Farms
Debbarma et al. (2021)	L	Capital, Energy, Labor	Production (Euro)	Agricultural sector
P. Singh, Singh, Sodhi, and Benbi (2021)	L	Biocides, Energy, Machinery, Seed, Fertilizers	Production, Production (Euro)	Rice, Wheat, rotation
M. Zhang et al. (2021)	I	Ferilizers, Irrigation, Land, Machinery	Agricultural Output, CO_2_ emissions	Agricultural sector
Streimikis et al. (2021)	I	Energy consumption, Labor, Land, fixed consumption	Purchasing Power Parity	Agricultural sector
Payandeh et al. (2021)	L	Agro-chemicals, Diesel fuel, Human labor, Machinery, Oil, Seeds, Fertilizers	Grain Yield, Yield (Straw)	barley
Güney (2021)	L	Fuel, Labor, Machinery, Pesticides, Seed, Fertilizers	Production	Wheat
Kyrgiakos et al. (2021a)	L	Energy, Irrigation, Labor, Land, PNPs, Seeds	Production, Production (Euro), Social characteristics	Cotton
Basavalingaiah et al. (2022)	L	Agro-chemicals, Diesel, Ferilizers, Labor, Lime, Machinery, Manure	Coffee, Yield	Coffee
Pan et al. (2021)	N	Energy, Land, Technolgy, funds, resources	Production	Agricultural sector
Lamkowsky et al. (2021)	L	Capital, Feed, Labor, Land, Machinery, Number of animals, Pesticides, Seeds, Veterinary costs, Fertilizers	Dairy Sales, Other Sales	Dairy farms
P. Singh, Singh, Sodhi, and Sharma (2021)	L	Biocides, Diesel, Electricity, Fuel, Irrigation, Labor, Seed, Fertilizers	Production	Wheat
Banaś et al. (2021)	L	Area, Labor, Land, Logging costs, Other costs, Protection costs, Silviculture, Standing volume	Production	Timber
Martinsson and Hansson (2021)	N	Energy expenditures	Contribution to global warming	Dairy farms
Y. Zhang et al. (2021)	I	CO_2_ Emissions, Land, Yield, food loses, protein supply	Agricultural added value, import dependency ratio, irrigated area	Agricultural sector
Nirmal Ravi Kumar and Babu (2021)	L	Manure, Seeds, gypsum	Production	Groundnut
Mwambo et al. (2021)	L	Agricultural Services, Evapotranspiration, Human labor, Seeds, animal Labor, Fertilizers, topsoil loss	Yield	Maize
J. Li et al. (2021 )	N	Labor, Land, Machinery, Fertilizers	Agricultural added value	Agricultural sector
Bagheri (2021)	I	Electricity, Labor	CO_2_ emissions, GDP	Agricultural sector
Seo and Umeda (2021)	L	Pest control cost	Gross farm income, Surplus of working hours	Rice
Tang et al. (2022)	N	Irrigation, Labor, Land, Machinery, Pesticides, Plastic film, Fertilizers	Economic output, Environmental output, Social output	Agricultural sector
P. Xu et al. (2022)	N	Agro-chemicals, Area, Electricity, Irrigation, Labor, Machinery	Output value	Agricultural sector
Guth et al. (2022)	N	Capital, Labor, Land	Production	Agricultural sector
Bernard et al. (2022)	I	Capital, Labor, Land	Agricultural added value	Agricultural sector
Ramos de Oliveira et al. (2022)	N	Diesel, Fleet condition, Fuel consumption, Gini index, Production costs, Warehouse capacity, photochemical oxidant formation, transportation matrix	GHG emissions, Wheat, logistic cost	Logistics
Streimikis et al. (2022)	I	Capital, Energy, Labor, Land	Net income	Agricultural sector
S. Wang et al. (2022)	P	Labor, Land, Blue Footprint, Green Footprint	Yield	Agricultural sector
Sharma (2022)	I	Land, Seeds, Fertilizers	Production	Rice
Yan et al. (2022)	L	Agro-chemicals, Irrigation, Labor, Land, Machinery	Production	Grain
Kord et al. (2022)	N	Irrigation, Labor, Land	Net income, Yield	Agricultural sector
Ziętek-Kwaśniewska et al. (2022)	P	Depreciation expenses, Electricity, Labor, Other costs, Raw materials	Net sales revenue	Dairy farms
Chaubey et al. (2022)	P	Area, GDP, Labor, Population, percipitation	Production	Agricultural sector
W. Li et al. (2022)	N	Capital, Labor, Land	Net income	Agricultural farms
Gao et al. (2022)	N	FamilyLabor, Hired Labor, Irrigation, Machinery, Manure, Pesticides, Seeds, Fertilizers	Yield	Rice
Chang et al. (2022)	N	Hired Labor, Irrigation, Machinery, Pesticides, Seed, Seeds, Fertilizers	Production	Agricultural sector
Ramezani et al. (2022)	L	Agro-chemicals, Irrigation, Labor, Land, Manure, Nitrogen balance	Yield	Saffron
Mohammadi et al. (2022)	L	Agro-chemicals, Biosides, Diesel, Electricity, Irrigation, Labor, Machinery, Seeds, Urea	Yield	Wheat
Abbas et al. (2022)	L	Biocides, Diesel, Irrigation, Labor, Machinery, Manure, Pesticide risk, Pesticides, Phosphorus balance, Seed, Fertilizers	Yield	Cotton
Turner et al. (2022)	L	Feed, pullets	Eggs	egg farms
L. Yang et al. (2022)	P	Diesel, Ferilizers, Pesticides	Income, Yield	Sugarcane
Khan and Ali (2022)	N	Land	Yield	Clover
Nyamuhirwa et al. (2022)	L	Capital, Labor	Added Value	Agricultural sector
Sui et al. (2022)	P	Capital, Labor, Land	Yield	Garlic
Wu et al. (2022)	N	Agricultural film input, Expected output, Irrigation, Labor, Land, Machinery, Pesticides, Fertilizers	Carbon emissions, Net income, Non point sources, Output value	Agricultural sector
Nodin et al. (2022)	P	Agro-chemicals, Capital, Labor, Land	Yield	Rice
Dania et al. (2022)	L	Collabotation Factors	Economic, Environmental, Social	Agricultural sector
Lu et al.(2022)	N	(3 Stage NDEA model with circular flows)		Agricultural sector

^*^Applicability level: Local (L), Province (P), National (N), International (I), Theoretical Model (T.M).

^**^ Output section does not include undesirable outputs.
